# Editorial

**Published:** 1891-04

**Authors:** 


### Editorial.

### TO MY JUNIOR BRETHREN.

To abandon old and tried friends, and seek new ones, often less worthy, to fill their places, seems to be an inherent weakness in mankind. It denotes want of sincerity and lack of stability. We are so constituted that we love change and devaricate from familiar paths to gratify foolish whims. We love the mysterious and miraculous; something novel and divergent from the old way. The mind, naturally, is fond of romance. There are those who live in fiction, and spend their time in rearing air castles. Nothing substantial is enjoyed by them. They do not like the solid realities of life, and therefore are unsettled and visionary. They yield to the charms of novelty, which creates a sort of inspiration, that captivates and delights, just because it is novel. This affords temporary pleasure, engenders new tastes and begets new aspirations. The stern demands of necessity help to brush away these gossamers from the mind, and replace them by pictures taken from the real civilization, leads us away from the darkness, the errors and wrong beliefs of former ages, and gives light and glory to the present—And through it is a kind of alchemy, changing baser and courser customs and laws into more liberal, humane and protective regulations and statutes, it has not been able to erect an unerring standard for individuals and governments for their guidance. Let us then act according to the clearest lights before us; accept nothing outside the realms of reason and enlightened experience. Let us examine our mooring and see if the anchors are fastened to solid and enduring foundations; dangers may be averted, life may be saved. This is an age of utilitarianism. Practical thought has been developed and cryttalized into the grandest structures of art. The chasing of the butterfly, the following of the Jack O'lantern, is gradually becoming a thing of the past. Wise and deliberate opinions, properly founded, should guide and direct us in our professional pursuits. The exercise of the soundest judgment in the most thorough analysis of facts, should govern us in our investigations. Sift the true from the false.

Weigh everything on the accurate balances of genuine logic, and all will be well. Let us not madly rush into unsettled and doubtful positions because of their novelty, nor blindly cling to doctrines and practices which have little but age to commend them. We should be considerate and conservative, and think more for ourselves. Brown-Sequard's elixir vitae was not a success, though it had his great name to support it. He was a faithful and honest experimentor, for he tried it on himself. To rejuvenate the old was a noble undertaking, and I am sorry for the failure. Something may come of it yet. Who knows? Let us hope.

Of late the secular press has been heralding accounts of what many suppose to be the grandest discovery of the century. The medical press has given but scant and unsatisfactory notice, and therefore we have had our fears, but have waited developments. There has been a wild rush by the professional and non-professional to give introduction to the Princess Lympha. She has not yet made her formal debut, and we will not intrude while attendants are arranging her toilet. We hope that, divested of the meretricious attire first assumed, she will come forth clad in the royal robe of honorable medicine.

A. W. G.

### GEORGIA STATE MEDICAL ASSOCIATION.

Please allow me to announce in your Journal that the Medical Association of Georgia will hold its forty-second annual session in the beautiful city of Augusta, April 15th to 17th, inclusive, prox.

A letter just received from the Chairman of the Programme Committee, assures me that there will be an attractive and interesting programme for the occasion. The fact that the same committee, who gave us such an interesting programme at Brunswick last year, is engaged to arrange the programme for this year, is a sufficient guarantee that we will have a pleasant and profitable meeting. With the indefatigable Foster as chairman, aided by the enterprising and plucky Lancaster and Holmes, we may rest assured of a fine programme, and a profitable session. Dr. Foster writes me that the profession of Augusta have their arms and hearts wide open to give the Association a cordial welcome, and that the City will entertain her guests handsomely. That full and ample hotel accommodations can be had. And that they are very anxious to have a full attendance. He asks that I urgently invite every graduate of a regular medical college to attend and become a member Reduced railroad rates have been secured an all railroads in Georgia upon the certificate plan. Be sure to get from the agent, where the going ticket is purchased, a certificate of such purchase to attend the meeting, and this certificate, when signed by the Secretary of the meeting, will entitle the holder to return on one third of the usual fare. If the ticket is bought at an intermediate station, at which through tickets to Augusta cannot be purchased, buy to Atlanta, Macon, Albany, Savannah, Athens or Americus, as may be most convenient, and repurchase to Augusta, taking certificates from both agents from whom tickets are secured. The certificate obtained at the above named points will be honored at Augusta, returning to the point at which it was secured, and the other certificate will be honored from thence to the starting point. These tickets good until the 20th, inclusive.

It is very desirable to make this the largest and most profitable session in the history of the Association, and the Association extends a cordial invitation to every graduate of a regular medical college, in good standing, to attend the approaching meeting and become a member.

Respectfully and fraternally,

Macon, Ga. K. P. MOORE, Secretary.

### MEETING OF THE NATIONAL ASSOCIATION OF RAILWAY SURGEONS.

At the Kansas City meeting of the National Association of Railway surgeons last year, it was decided to hold the next meeting at Buffalo, May 7th, 8th and 9th of this year. But on account of the meeting of the American Medical Association being set for the same time, it has been decided to change those dates, and to hold our next meeting at Buffalo April 30th and May 1st and 2d, to which all Railway Surgeons are cordially invited. To all Railway Surgeons sending their names and addresses to the corresponding Secretary, a copy of the Constitution and Programme will be sent. All those wishing to read papers should send in the titles of their papers without delay. For further information inquire of

A. G. GUMAER, M. D.,
Corresponding Secretary,
Buffalo, N. Y.

---

### UNCONSCIOUS PARTURITION IN A PRIMIPARA.

A case of high obstetric and medico-legal interest is to be found in the *Archives de Tocologie* for November. Physiologically painless parturition is rare. Tarnier has related some cases, including one instance where a Canadian woman occasionally dropped a baby on the ground, at term, without noticing it. In Howard's case, labor took two hours; the patient was reading a book till a quarter of an hour before the child was delivered, which event occurred after some straining, not sufficient to make her cry out. In Dr. Brunon's case, newly reported, a married woman, aged 22, had a troublesome cough one day shortly before term. The coughing was accompanied with lumbar pains, which increased. At 11 o'clock in the evening the patient tried to pass a motion. She sat over one hour in the closet, believing that her pains signified painful defecation. Then she went to bed. At half past 1 o'clock she woke up feeling a desire to pass a motion, with lumbar pains, such as she had felt before when constipated. As she rose to go to stool a smart lumbar pain occurred, and she felt something between her thighs. On handling it she found, much to her surprise, that it was the head of her first-born. She declared to Dr. Brunon that the pains were entirely lumbar, she had no colicky sensations, and none of the expulsive pains usually so severe, especially in primiparæ. The desire to defecate was strong, and she stated that the child might have been born into the pan of the closet without her recognizing the truth of her condition till the moment of its delivery. The patient was an intelligent, well educated woman, free from any neurosis. This case proves that in the case of an experienced person an infant might be expelled into the water in the pan of a closet without intended infanticide.—*Brit. Med. Journal.*

